# Chiral capillary electrophoresis with UV-excited fluorescence detection for the enantioselective analysis of 9-fluorenylmethoxycarbonyl-derivatized amino acids

**DOI:** 10.1007/s00216-018-1148-x

**Published:** 2018-05-29

**Authors:** Amir Prior, Giulia Coliva, Gerhardus J. de Jong, Govert W. Somsen

**Affiliations:** 1 Division of BioAnalytical Chemistry, Amsterdam Institute for Molecules, Medicines and Systems, Vrije Universiteit Amsterdam, de Boelelaan 1085, 1081 HV Amsterdam, The Netherlands; 2 Biomolecular Analysis, Utrecht University, Universiteitsweg 99, 3584 CG Utrecht, The Netherlands

**Keywords:** Amino acids, Chiral separation, Capillary electrophoresis, FMOC derivatization, Fluorescence detection, Cerebrospinal fluid

## Abstract

The potential of capillary electrophoresis (CE) with ultraviolet (UV)-excited fluorescence detection for sensitive chiral analysis of amino acids (AAs) was investigated. dl-AAs were derivatized with 9-fluorenylmethoxycarbonyl chloride (FMOC)-Cl to allow their fluorescence detection and enhance enantioseparation. Fluorescence detection was achieved employing optical fibers, leading UV excitation light (< 300 nm) from a Xe-Hg lamp to the capillary window, and fluorescence emission to a spectrograph equipped with a charge-coupled device (CCD). Signal averaging over time and emission wavelength intervals was carried out to improve the signal-to-noise ratio of the FMOC-AAs. A background electrolyte (BGE) of 40 mM sodium tetraborate (pH 9.5), containing 15% isopropanol (*v*/*v*), 30 mM sodium dodecyl sulfate (SDS), and 30 mM β-cyclodextrin (β-CD), was found optimal for AA chemo- and enantioseparation. Enantioresolutions of 1.0 or higher were achieved for 16 proteinogenic dl-AAs. Limits of detection (LODs) were in the 10–100-nM range (injected concentration) for the d-AA enantiomers, except for FMOC-d-tryptophan (536 nM) which showed intramolecular fluorescence quenching. Linearity (*R*^2^ > 0.997) and repeatability for peak height (relative standard deviations (RSDs) < 7.0%; *n* = 5) and electrophoretic mobility (RSDs < 0.6%; *n* = 5) of individual AA enantiomers were established for chiral analysis of dl-AA mixtures. The applicability of the method was investigated by the analysis of cerebrospinal fluid (CSF). Next to l-AAs, endogenous levels of d-glutamine and d-aspartic acid could be measured in CSF revealing enantiomeric ratios of 0.35 and 19.6%, respectively. This indicates the method’s potential for the analysis of low concentrations of d-AAs in presence of abundant l-AAs.

## Introduction

Amino acids (AAs) play a major role in the physiology of organisms being building units of proteins but also essential in, e.g., metabolic processes, neurotransmission, and lipid transport. AAs are precursors for the synthesis of hormones and low-molecular-weight nitrogenous substances [1, 2]. Most AAs in human life are chiral and predominantly occur in their l-form. The presence of free d-AAs in the brain of humans and other mammals has been known for many years but for long time was thought to originate from bacteria [3] or formed by spontaneous racemization of l-AAs [4]. More recently, endogenous racemases were shown to be involved in d-AA synthesis [5, 6]. Some d-AAs were found in relatively large quantities during the embryonic phase of brain development [7], localized to protoplasmic astrocytes, or closely distributed to NMDA receptors [8, 9], which suggests their function in signaling pathways, neurotransmission, and in brain biology [10]. d-AAs were found to have essential roles and abnormal d-AA levels and AA enantiomeric ratios in cerebrospinal fluid (CSF) may relate to neurodegeneration. Indeed, d-AAs were found to be involved in the pathogenesis of psychiatric diseases and abnormal levels were associated with human disorders, such as schizophrenia and Alzheimer’s disease [11–15]. For example, Samakashvili et al. [16] showed that chiral analysis of AAs in CSF might be useful for the early diagnosis and understanding of metabolism processes related to neurodegeneration and Alzheimer’s disease. Clearly, analysis of d-AAs and determination of the d/l ratios can be of importance in clinical and pharmaceutical science but also in environmental and food analysis [17–20]. AA enantiomers of interest often are minor components of multi-component complex samples, such as tissues and biological fluids, requiring both chemo- and enantioselective separation with sensitive detection in order to allow their unambiguous assessment.

Chiral analysis of AAs can be performed by gas chromatography [21] and high-performance liquid chromatography [22, 23], requiring costly chiral stationary phases and/or relatively large amounts of (chiral) derivatization agents [24–27]. In addition, upon repeated analysis of biological samples, lifetimes of chiral columns may be limited. Capillary electrophoresis (CE) has shown to be a versatile alternative tool for the enantioselective analysis of AAs requiring minute sample volumes and small amounts of solvents and chiral selector molecules. These selectors, such as (derivatized) cyclodextrins (CDs) [28, 29], vancomycin [30, 31], and 18-crown-6-tetracarboxylic acid [32], can be simply added to the background electrolyte (BGE). A limitation of CE is the relatively low concentration sensitivity obtained with common ultraviolet (UV) absorbance detection, due to the small optical path length provided by the capillary internal diameter. Besides, only the aromatic AAs tryptophan, tyrosine, and phenylalanine show native UV absorbance. Therefore, AAs are often labeled with UV or visible light-absorbing agents and subsequently analyzed by CE with UV or fluorescence (Flu) detection [33–36]. The use of derivatization agents will not only improve detectability and sensitivity but also detection selectivity as only analytes with specific reactive groups will be derivatized and thus detected. Furthermore, derivatization of AAs may also enhance enantioseparation [37].

Chiral CE analysis of AAs with fluorescence detection has predominantly been done employing fluorescein isothiocyanate (FITC) [16, 33, 34, 38–40] but also, e.g., naphthalene-2,3-dicarboxyaldehyde (NDA) [41], 4-fluoro-7-nitro-2,1,3-benzoxadiazole (NBD-F) [42], 5-(4,6-dichloro-s-triazin-2-ylamino) fluorescein (DTAF) [43], and 5-carboxyfluorescein succinimidyl ester (CFSE) [44] have been used for derivatization. These reagents, however, require relatively long derivatization times (30 min to several hours). More rapid derivatization (few minutes) can be achieved with 9-fluorenylmethoxycarbonyl chloride (FMOC-Cl). Under alkaline conditions, FMOC reacts with primary and secondary amines and allows fast derivatization of all proteinogenic AAs [45]. Chiral CE-UV of FMOC-derivatized AAs has been described [45–48]. Although FMOC is fluorescent, and thus would allow more sensitive fluorescence detection, chiral CE-Flu of FMOC-derivatized AAs has not been reported so far. This is most probably due to the fact that FMOC requires excitation in the UV region, which is not provided by conventional CE-Flu systems employing lasers with output in the visible region. Chan et al. [49] used a home-built laser-induced fluorescence (LIF) detector for the CE analysis of FMOC-AAs but did not perform chiral separations. In CE, UV-excited fluorescence detection of other compounds (e.g., vitamins, drugs, phenolic compounds, and proteins) has been achieved using a laser source (mostly 266 nm) but also lamp-based excitation using a Xe-Hg light source (for excitation in the range of 220–400 nm) has been reported using a dedicated detection cell [50–55]. The latter showed up to 160-fold sensitivity improvement as compared to UV absorbance detection, whereas sensitivity was comparable as obtained with LIF detection, while providing much higher flexibility in selection of UV excitation wavelengths.

In the present paper, we studied chiral CE-Flu of FMOC-derivatized AAs employing lamp-based UV excitation. Using a CE-dedicated fluorescence setup [50–55], the light from a Xe-Hg excitation lamp is led to the separation capillary by an optical fiber and focused onto the detection window. The analyte emission light is partly trapped within the fused silica capillary and guided along the capillary by total internal reflection. The fluorescence light is coupled out of the capillary by an optical cone and directed via a liquid light guide towards the detector, which was comprised of a spectrograph with a charge-coupled device (CCD) detector. Fluorescence excitation and emission parameters were studied in order to achieve optimal sensitivity of FMOC-AAs. Separation conditions were investigated and optimized for chiral and chemical resolution. Analytical aspects of the CE-Flu method, such as repeatability, linearity, and detection limits, were evaluated. The method’s applicability was studied by the enantioselective analysis of AAs in CSF.

## Materials and methods

### Chemicals

All reagents were of analytical grade. FMOC-Cl, β-CD, pentane, sodium tetraborate, sodium hydroxide, glycine, d-glutamic acid, d-histidine, d-threonine, l-alanine, l-arginine, l-asparagine, l-aspartic acid, l-cysteine, l-glutamic acid, l-glutamine, l-histidine, l-isoleucine, l-leucine, l-lysine, l-methionine, l-proline, l-serine, l-threonine, l-tryptophan, l-tyrosine and l-valine, dl-alanine, dl-arginine, dl-asparagine, dl-aspartic acid, dl-cysteine, dl-glutamic acid, dl-histidine, dl-isoleucine, dl-leucine, dl-lysine, dl-phenylalanine, dl-proline, dl-serine, dl-tryptophan, and dl-valine were from Sigma-Aldrich (Steinheim, Germany). Isopropanol, dl-methionine, dl-tyrosine, sodium dodecyl sulfate, and acetonitrile were supplied by Fluka (Steinheim, Germany). Water was deionized and purified with a Milli-Q purification system (Millipore, Belford, NJ, USA).

The optimal BGE was 40 mM sodium tetraborate (adjusted to pH 9.5 with 1 M sodium hydroxide) containing 15% (*v*/*v*) isopropanol, 30 mM sodium dodecyl sulfate (SDS), and 30 mM β-CD. The BGE was filtered prior to use through 0.45-μm pore size disposable nylon filters from VWR (Amsterdam, The Netherlands). Stock solutions (3 mM) of AAs were prepared in 0.2 M sodium tetraborate (pH 9.5).

### Derivatization

The pH of the CSF samples was adjusted by adding 10 μL of 2 M sodium hydroxide to 990 μL CSF.

Derivatization of AAs with FMOC was carried out as described earlier [47]. Briefly, 500 μL of 10 mM FMOC in acetonitrile was added to 500 μL sample (i.e., ≤ 3 mM AA in 0.2 M sodium tetraborate buffer (pH 9.5) or pH-adjusted CSF). This mixture was kept at room temperature for 2 min and then extracted with 1.5 mL pentane to remove excess of FMOC reagent. The aqueous phase was diluted ten times with water. The resulting solution was kept at 4 °C until injection.

### CE-Flu system

CE experiments were carried out with a P/ACE MDQ CE instrument (Beckman Coulter, Brea, CA, USA). CE of AAs was performed using bare-fused silica capillaries (Polymicro Technologies, Phoenix, AZ, USA). The capillaries had an i.d. of 75 μm, an o.d. of 375 μm, and total/effective lengths of 72.2/55.3 cm. The capillary temperature was set to 22 °C. New bare-fused silica capillaries were rinsed with 1 M sodium hydroxide for 10 min at 30 psi and deionized water for 10 min at 30 psi. Between CE analyses, the capillaries were rinsed with BGE for 5 min at 30 psi. Overnight, the capillaries were stored in deionized water. Separations were performed in normal polarity mode with a separation voltage of 25 kV. Sample injection was performed hydrodynamically by applying 0.5 psi for 13 s, which corresponds to an injected volume of about 0.8% of the total capillary volume (BGE viscosity relatively to water = 1.93). Data acquisition was performed using 32 Karat software (Beckman Coulter).

A previously described wavelength-resolved fluorescence (wrFlu) detector for CE was used, which was based on an Argos 250B fluorescence detection cell (Flux Instruments, Basel, Switzerland) [55] combined with a SR-163 spectrograph equipped with a CCD camera (Andor Technologies, Darmstadt, Germany) [56, 57]. The Argos system comprises a Xe-Hg lamp for excitation, excitation, and emission optical guides and filters and an optical cone detection cell. A capillary cartridge with an external detector adapter (Beckman Coulter) was used to guide the CE capillary from the inlet vial out of the CE instrument through the Argos fluorescence detector cell towards an external outlet vial containing the grounding electrode. The external detector adapter guarantees undisturbed capillary cooling by facilitating the CE instrument’s liquid cooling. The emission light optical guide was connected to the spectrograph via a home-made fiber holder equipped with a back illuminated CCD chip of 256 × 1024 pixels with a pixel size of 26 μm^2^ (Andor Technologies). The spectrograph comprised a grating of 600 lines/mm blazed at 300 nm and a band-pass of 263 nm. The CCD chip was cooled down to − 60 °C. The spectrograph was wavelength-calibrated daily using the reference spectral lines of an Hg pen-ray light source (L.O.T.-Oriel, Darmstadt, Germany). Typical detection settings used in CE-Flu experiments were slit width, 50 μm; exposure time, 3 s; vertical shift speed, 16.25 μs; and horizontal read-out rate, 33 kHz. Acquired spectra were collected using the Full Vertical Binning mode and were background corrected. A 300-nm short-pass interference filter (Asahi Spectra USA Inc., Torrance, CA, USA) was used to select excitation light. Other tested excitation filters were a 260 (± 10)-nm band-pass filter (Asahi Spectra) and a 240–400-nm broad-pass filter (Flux Instruments). Data acquisition analysis was performed using the software program Andor Solis (Andor Technologies).

### Fluorescence spectra

Reference excitation and emission spectra of FMOC-AAs (10 μM in water) were recorded using an LS 50B fluorescence spectrometer (PerkinElmer, Waltham, MA) at room temperature using excitation and emission slit widths of 15 and 4 nm, respectively, and a scan rate of 3 nm/s.

### Statement of human and animal rights

No human or animal subjects were used in this study.

## Results and discussion

### Fluorescence detection of FMOC-AAs

Based on previous studies [29, 48, 58], a BGE of 40 mM sodium tetraborate (pH 9.5) with 15% (*v*/*v*) isopropanol and 30 mM β-CD was selected as a starting condition to investigate the fluorescence detection of AA enantiomers. A test mixture of the dl-AAs alanine, aspartic acid, glutamic acid, leucine, methionine, and tryptophan was derivatized with FMOC-Cl. These AAs represent diverse chemical properties and exhibit different overall charge after derivatization. A bare-fused silica capillary with an i.d. of 75 μm was used in order to maximize the optical path length for excitation, without inducing excessive CE current. Preliminary CE-UV experiments showed that under these conditions all test AAs were separated with an enantioresolutions ranging from 0.7 for alanine to 6.4 for glutamic acid.

**Fig. 1:**
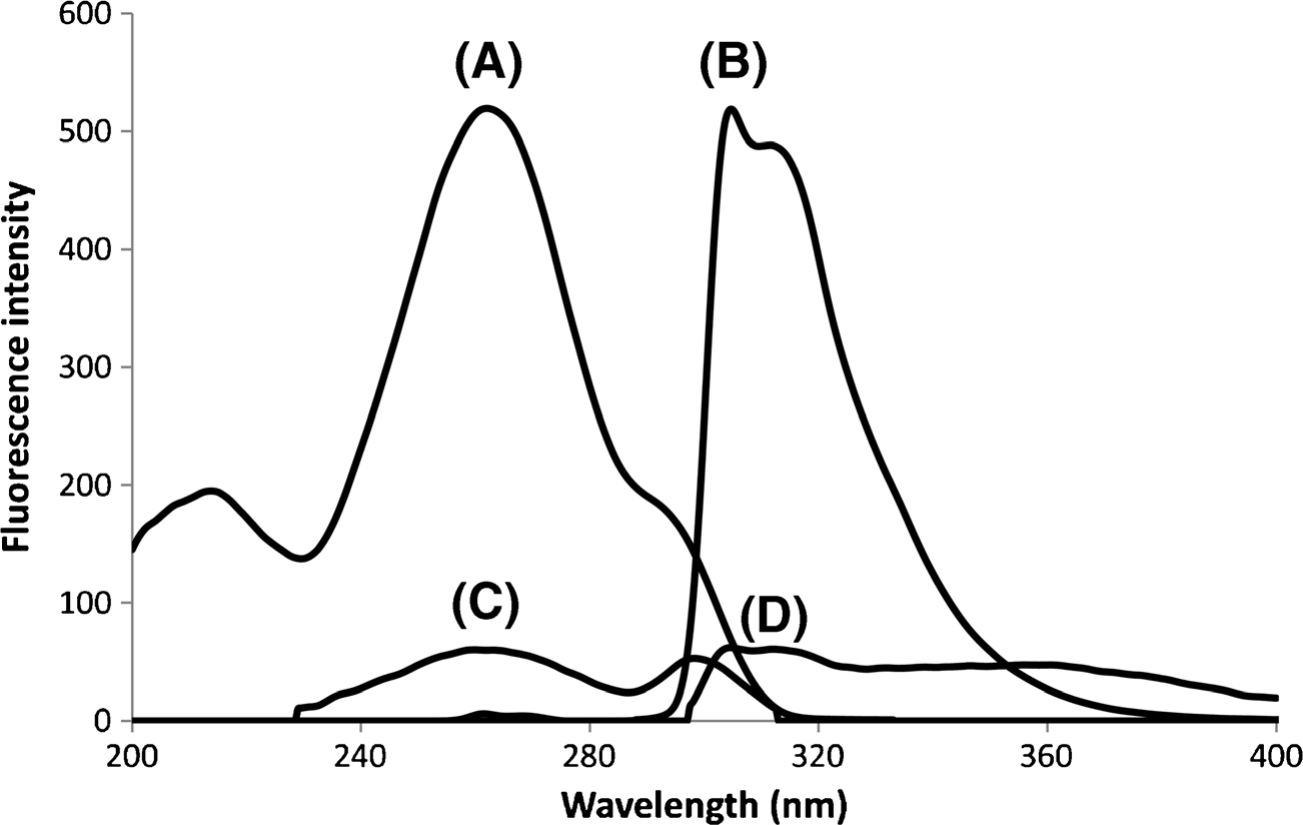
Excitation (**A**, **C**) and emission (**B**, **D**) spectra of FMOC-AAs (10 μM) in water recorded with a standalone fluorescence spectrophotometer. (A + B) FMOC-phenylalanine. (C + D) FMOC-tryptophan. Experimental conditions, see section “Materials and methods”

The excitation and emission spectrum of FMOC-dl-phenylalanine was recorded using a conventional spectrofluorometer (Fig. 1A and B), clearly indicating that FMOC-AAs require excitation in the deep UV region for efficient fluorescence measurement. In order to achieve appropriate UV excitation, a CE-dedicated fluorescence detector equipped with a Xe-Hg source was employed [55]. Previously, this system has shown useful for measuring native protein emission upon UV excitation [57]. The system comprises a spectrograph and CCD allowing on-line wavelength-resolved fluorescence (wrFlu) detection. The emission spectrum of FMOC-dl-phenylalanine recorded with wrFlu differed somewhat from the reference spectrum (Fig. 1B) showing a fluorescence maximum at 331 nm. The difference in spectral shape and maximum wavelength is caused by a reduced transmittance for UV wavelengths below 315 nm of the detector optics (optical cone and emission light fiber) [57].

In order to achieve optimum detection of FMOC-AAs, excitation conditions were varied testing a 260-nm band-pass filter, a 300-nm cutoff short-pass filter, and a 240–400-nm broad-pass filter. A sample of FMOC-dl-aspartic acid (1 μM) was repeatedly analyzed using the different excitation filters and the abovementioned BGE, which provided a resolution of 3.7 for the aspartic acid enantiomers. The signal-to-noise ratio (*S*/*N*) obtained for each enantiomer at an emission wavelength of 331 nm was determined (Table 1). The lowest *S*/*N* was observed using the 260-nm band-pass excitation filter. Noise levels were relatively low with this filter, but absolute signal intensities were modest, as only a part of the excitation spectrum is employed to induce FMOC-aspartic acid fluorescence. Creating a broader band-pass by using the 300-nm cutoff short-pass filter or the 240–400-nm band-pass filter, an increase of *S*/*N* was observed with respect to the 260-nm filter. With the 240–400 nm, much broader excitation indeed was achieved; however, it also significantly attenuated the overall excitation light intensity. Best *S*/*N* was obtained by using only the 300-nm cutoff short-pass filter for excitation. Although noise levels significantly increased, absolute signal intensities were nine times higher as obtained with the 260-nm band-pass filter, leading to most favorable detection of the FMOC-AA fluorescence.

**Table 1:** *S*/*N*s of FMOC-dl-aspartic acid (1 μM per enantiomer) obtained during CE-Flu using different excitation filters

Enantiomer	Excitation filter		
	260 nm	240–400 nm	< 300 nm
d-Aspartic acid	67.0	81.0	117.9
l-Aspartic acid	68.7	81.7	117.1

Experimental conditions: emission wavelength, 331 nm; for further conditions, see section “Materials and methods”

**Fig. 2:**
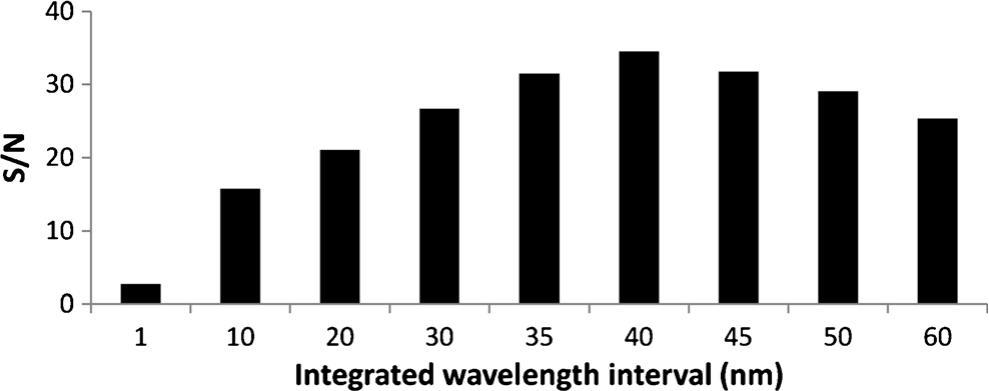
*S*/*N* as function of the integrated wavelength interval obtained during CE-Flu of 1.25 μM FMOC-d-aspartic acid. For experimental conditions, see section “Materials and methods”

**Fig. 3:**
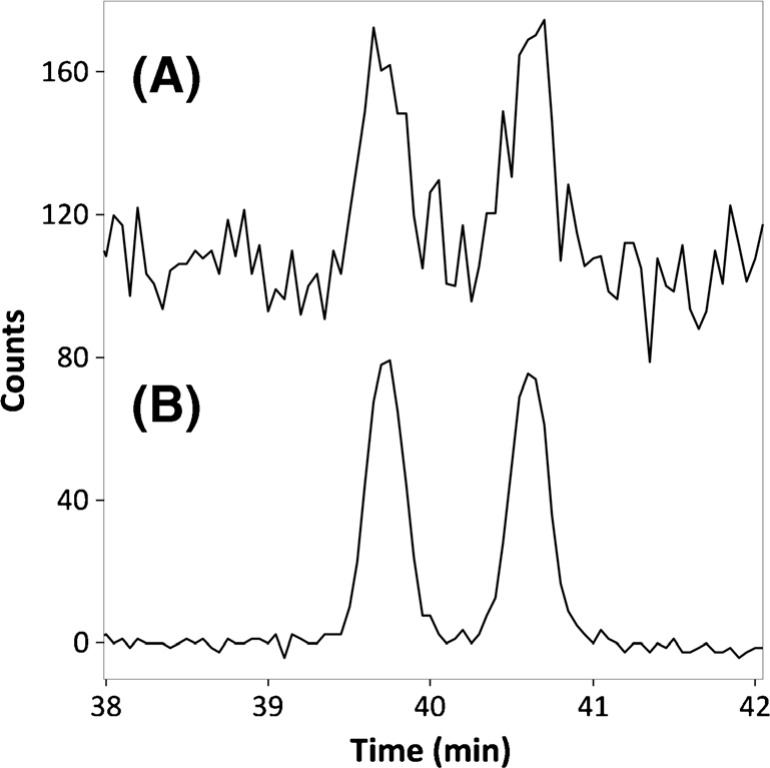
Electropherograms obtained during chiral CE-Flu of FMOC-dl-aspartic acid. (**A**) extracted electropherogram at an emission wavelength of 331 nm and (**B**) extracted electropherogram using emission signal averaging over 40-nm interval centered around 331 nm. Injected concentrations, 1.25 μM of each enantiomer; d-enantiomer migrates before l-enantiomer. For further experimental conditions, see section “Materials and methods”

The wrFlu detection provides the collection of a series of emission spectra over time. Using a detected emission wavelength of 331 nm, only a fraction of the measured emission is used to construct an electropherogram. Integration of recorded emission intensities over a certain wavelength range for every measured point in time might be used to increase *S*/*N* of the FMOC-AA signals. To evaluate this option, extracted electropherograms were constructed from the CE-Flu data obtained for FMOC-d-aspartic acid using the integrated signal of increasing wavelength intervals centered around 331 nm (Fig. 2). The *S*/*N* grows steadily with increasing wavelength interval, until it levels off at a width of about 40 nm. For wavelength intervals wider than 40 nm, the integrated signal intensity does not significantly increase, while the integrated noise increases proportionally, yielding a loss in *S*/*N*. The gain obtained with wavelength interval integration is clearly illustrated by Fig. 3, showing the analysis of FMOC-dl-aspartic acid (1.25 μM for each enantiomer). Using signal integration, the *S*/*N* increased with a factor of 12 with respect to single wavelength detection, leading to limits of detection (LODs) of less than 100 nM.

### Optimization of FMOC-AA separation

**Fig. 4:**
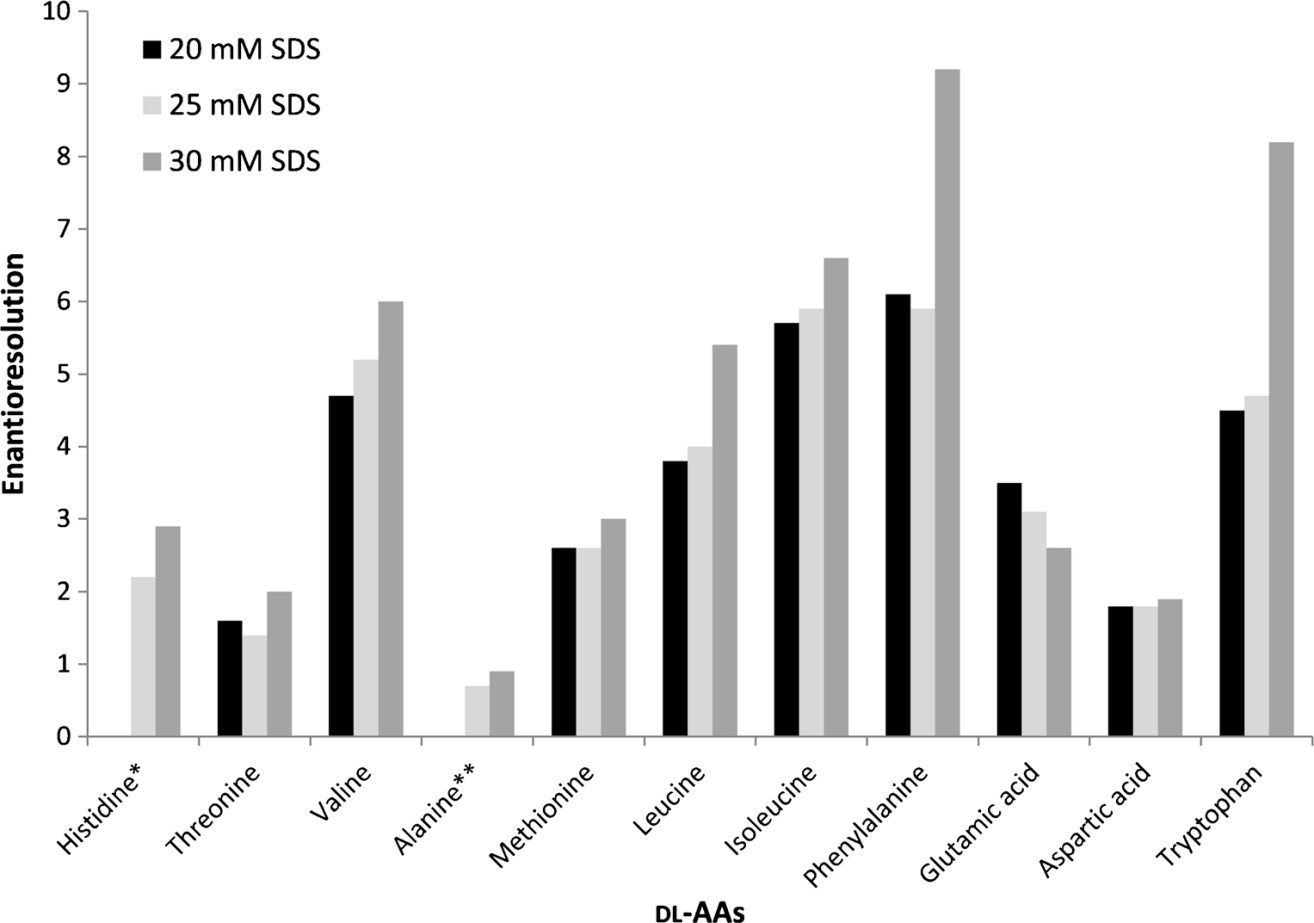
Effect of SDS concentration in the BGE on the FMOC-AA enantiomeric resolution. BGE, 40 mM sodium tetraborate (pH 9.5) containing 15% isopropanol, 30 mM β-CD and SDS. For further experimental conditions, see section “Materials and methods”. Asterisk: At 20 mM SDS in the BGE, the histidine enantiomers co-migrated with unreacted FMOC and could not be observed. Double asterisk: At 20 mM SDS in the BGE, the alanine enantiomers were not separated

In order to achieve enantioselective analysis of multiple AAs by CE-Flu, both chiral separation (i.e., enantioresolution) and mutual separation (i.e., chemoresolution) of the different AAs are required. Enhancement of AA separation can be attained by addition of SDS to the BGE, inducing micellar electrokinetic chromatography [29]. Therefore, a test mixture of 11 FMOC-dl-AAs (histidine, threonine, alanine, valine, methionine, isoleucine, glutamic acid, aspartic acid, leucine, phenylalanine, and tryptophan) was analyzed using BGEs of 40 mM sodium tetraborate (pH 9.5) with 15% (*v*/*v*) isopropanol, 30 mM β-CD, and 20, 25, or 30 mM SDS. Raising the SDS concentration from 20 to 30 mM resulted in longer analysis times (40, 47, and 83 min, respectively) but also in an up to three times higher resolution of the AAs and an overall enhancement of enantioresolution (Fig. 4). Indeed, use of SDS in the BGE resulted in significantly increased enantioseparation of FMOC-AAs in comparison with CE employing a buffer with only β-CD, as reported by us previously [58]. Highest enantioresolutions were observed at 30 mM SDS for most tested FMOC-AAs, except for aspartic acid that was not significantly affected by the SDS concentration and glutamic acid that showed a decrease of enantioresolution with increasing SDS concentration. FMOC-aspartic acid and FMOC-glutamic acid are doubly negatively charged and most probably cannot partition into the SDS micelles due to electrostatic repulsion [46]. A BGE concentration of 30 mM SDS was selected for further experiments.

The isopropanol content in the BGE was varied in the range of 13–17% in order to further fine-tune the separation of the 11 test FMOC-dl-AAs. Using 13% isopropanol in the BGE, the migration window of the tested FMOC-AAs was small, and as a result, many FMOC-AAs co-migrated. With 15 and 17% isopropanol in the BGE, the chemo- and enantioresolution improved significantly. With 17% isopropanol, 19 out of the 22 FMOC-AA enantiomers were mutually resolved, whereas with 15% isopropanol almost full separation was achieved with only l-aspartic acid and d-leucine co-migrating. The optimum BGE was 40 mM sodium tetraborate (pH 9.5) with 15% (*v*/*v*) isopropanol, 30 mM β-CD, and 30 mM SDS.

Finally, the effect of the capillary thermostating temperature (15–23 °C) on the analysis time and resolution was studied. Increasing the capillary temperature overall resulted in shorter migration times. For instance, at 15 °C, the migration time of dl-leucine was about 59 min, whereas at 23 °C, the enantiomers were detected after 44 min. Although for most AAs the enantioresolution slightly decreased with raising capillary temperatures, the chemoresolution of the AAs increased. As a compromise between analysis time, chemoresolution, and enantioresolution, a capillary temperature of 22 °C was selected.

**Fig. 5:**
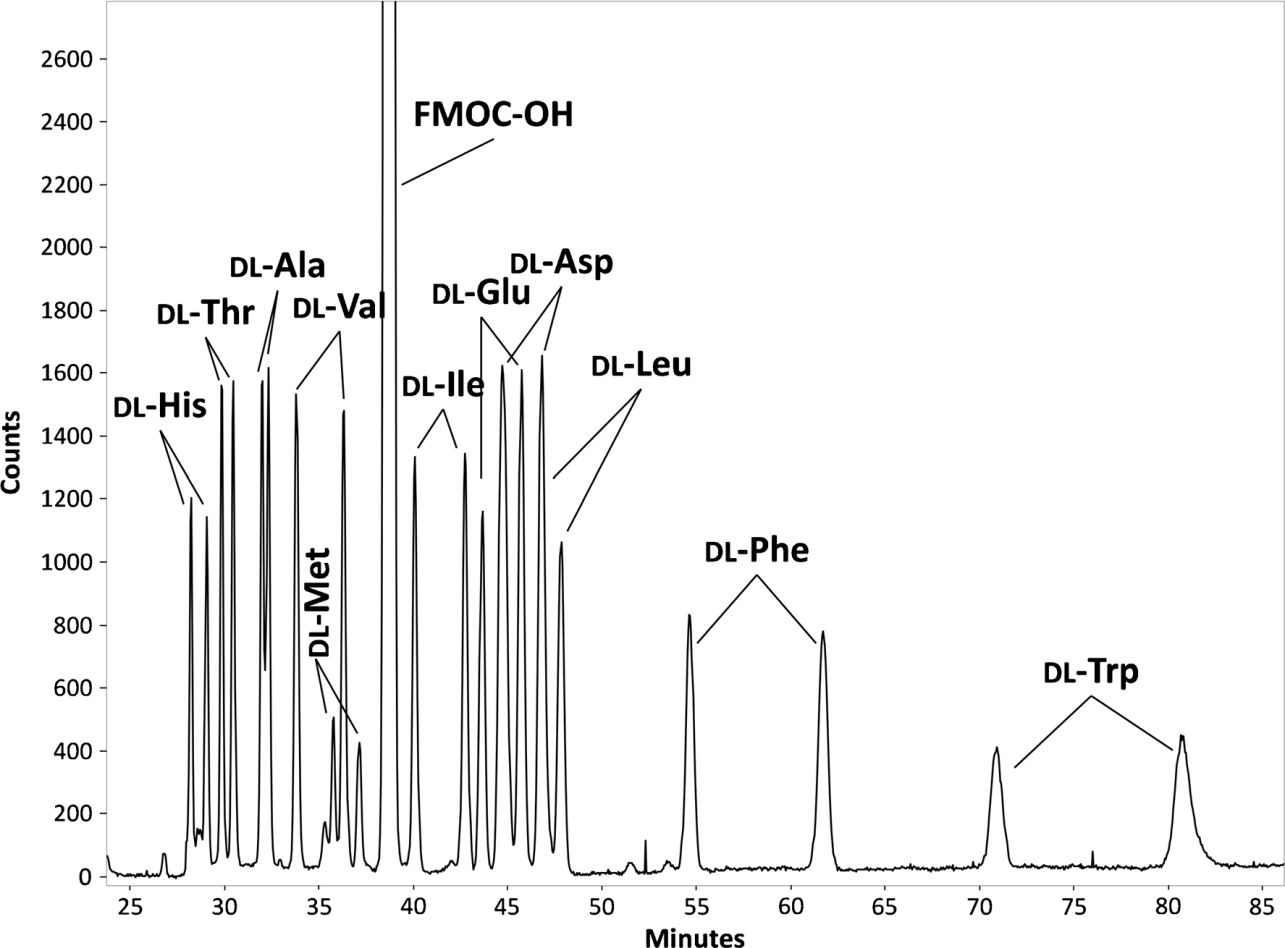
Electropherogram obtained during chiral CE-Flu of a mixture of 11 dl-AAs. For all FMOC-AAs, the d-form migrates before the l-form. Injected concentrations, 500 nM for each enantiomer, except for tryptophan, 5000 nM. For further experimental conditions, see section “Materials and methods”

Figure 5 shows the CE-Flu analysis of the 11 test dl-AAs derivatized with FMOC using the optimized method. All analyzed FMOC-AAs show enantioresolution (1.0–8.8) and are almost fully separated mutually. The system peak from the unreacted FMOC reagent is not interfering with the FMOC-AAs. Tryptophan and phenylalanine showed relatively long migration times, most probably due to their relatively high hydrophobicity and, therefore, strong interaction with the SDS micelles.

### Analytical performance

For the optimized CE-Flu method, precision of migration time and electrophoretic mobility were assessed by analyzing the 11 dl-AAs mixture in five consecutive runs, yielding relative standard deviations (RSDs) in the range of 1.6–5.8 and 0.1–0.6%, respectively. RSDs for peak height were in the range of 1.6–7.0%. Method linearity was assessed by derivatizing mixtures of dl-threonine and dl-leucine, which represent a fast and a slow migrating AA, of different concentration (75–3700 nM for each enantiomer). Good linearity was observed for the enantiomers of both AAs with coefficients of determination (*R*^2^) above 0.997.

The chiral performance and sensitivity of the optimized CE-Flu method were evaluated for 19 chiral proteinogenic AAs and glycine (Table 2). Tyrosine, lysine, and cysteine were not detected within 90 min of analysis. Lysine and cysteine—which carry two FMOC moieties after derivatization [29]—and tyrosine are quite hydrophobic and show high affinity for the SDS micelles, yielding very low mobility. Extending the analysis time revealed the enantioseparation of these AAs with migration times of up to 3.5 h. The other FMOC-AAs were successfully enantioseparated, showing resolution of 1.5 or higher, except for alanine (resolution, 1.0). The LODs (injected concentration yielding a *S*/*N* of 3) were in the range of 14–98 nM (2–15 ng/mL), except for the tryptophan enantiomers, which exhibited a LOD of 536 nM (110 ng/mL). The intensity of the FMOC-tryptophan excitation and emission spectra (Fig. 1C, D) indeed was lower than the intensity observed for the other FMOC-AAs (Fig. 1A, B) for the same concentration. The lower fluorescence yield of FMOC-tryptophan is due to intramolecular quenching of the FMOC emission by the indole moiety of tryptophan [59]. On average, the obtained LODs encompass an improvement of the sensitivity of two orders of magnitude when compared with chiral CE-UV methods for AAs using FMOC derivatization [45–48]. These studies reported LODs in the micromolar range.

**Table 2:** Enantiomer resolution and LODs (nM; ng/mL) obtained for 17 proteinogenic AAs using chiral CE-Flu

Amino acid^a^	Enantioresolution	LOD (nM)^b^	LOD (ng/mL)^b^
Alanine	1.0	22	1.9
Valine	7.4	19	2.2
Methionine	3.9	39	5.8
Threonine	2.2	21	2.5
Histidine	3.4	98	15.2
Isoleucine	5.4	52	6.8
Glutamic acid	3.8	19	2.8
Aspartic acid	1.9	27	3.6
Leucine	5.4	37	4.8
Phenylalanine	8.8	28	4.6
Tryptophan	7.1	536	109.4
Glycine	–	27	2.0
Proline	1.5	38	4.3
Serine	2.1	16	1.7
Asparagine	2.1	15	1.9
Glutamine	1.7	14	2.0
Arginine	3.4	36	6.2

^a^Injected concentration, 500 nM per enantiomer (except tryptophan, 5000 nM)

^b^Concentration yielding a *S*/*N* of 3 as calculated for the d-enantiomer

### Application to CSF

**Fig. 6:**
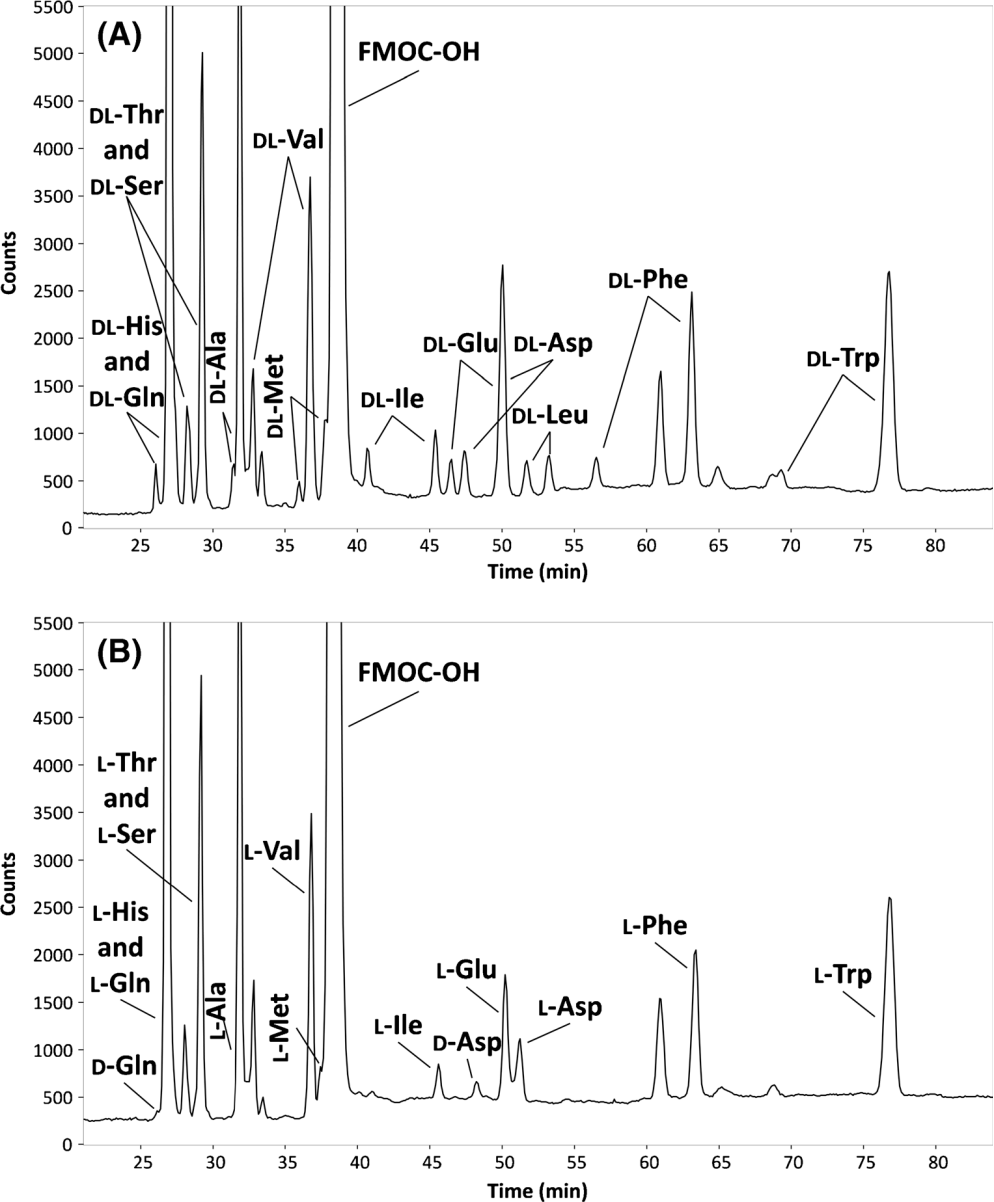
Electropherograms obtained during chiral CE-Flu of (**A**) CSF spiked with 13 dl-AAs, and (**B**) blank CSF. For (A) 5.00 μM per enantiomer was spiked into the CSF, except for tryptophan (50.0 μM), which corresponds to injected concentrations of 250 and 2500 nM, respectively. For further experimental conditions, see section “Materials and methods”

The feasibility of the developed chiral CE-Flu method for the detection of d-AAs in biofluids was investigated by the analysis of CSF. CSF was spiked with 13 dl-AAs at levels corresponding to a concentration of 250 nM in CSF for each enantiomer (except for tryptophan, 2500 nM) and analyzed by CE-Flu (Fig. 6A). Assignment of the peaks was performed by spiking CSF with individual FMOC-dl-AAs. All the tested FMOC-AAs could be detected in the CSF and each was enantioseparated. Nevertheless, for CSF, no chemoresolution of histidine and glutamine and of threonine and serine was achieved and the l-enantiomers of glutamic acid and aspartic acid co-migrated. Analysis of blank CSF (Fig. 6B) showed the natural presence of the l-enantiomers of glutamine, histidine, serine, threonine, alanine, valine, methionine, isoleucine, glutamic acid, aspartic acid, phenylalanine, and tryptophan. More importantly, the sensitivity of the CE-Flu method allowed direct detection of d-aspartic acid in the blank CSF (peak at 48 min in Fig. 6B). In addition, the small peak at 26 min (Fig. 6B) was assigned to d-glutamine as d-histidine is not expected to be present in CSF [11]. From the measured peak areas, the d/l-enantiomeric ratio of aspartic acid in CSF was calculated to be 19.6%. For glutamine, the d/l-enantiomeric ratio was estimated to be 0.35%. These enantiomeric ratios are within reported ranges for aspartic acid (18–25%) and glutamine (0.1–1.0%) in CSF [60, 61]. Based on the peak areas and the spiked concentrations, endogenous CSF levels for d-aspartic acid and d-glutamine of 1365 and 565 nM (182 and 82 ng/mL, respectively) were estimated, which is within reported ranges for these two d-AAs [61, 62]. In order to appreciate the LOD of the CE-Flu method for d-AA analysis in CSF, dl-leucine was selected as this AA was not present in the blank CSF analyzed. From the peak area obtained for the spiked CSF, the LOD for d-leucine in CSF was determined to be 1050 nM (138 ng/mL) which corresponds to an injected concentration of 52 nM (6.8 ng/mL) taking the dilution from the sample pretreatment into account. This value is similar to the LOD obtained for leucine in aqueous solution (Table 2), indicating minor effects of the CSF matrix on the analysis of the AAs. Overall, these results indicate the potential of the CE-Flu method to detect d-AAs next to their L-AA enantiomers in a biofluid.

The developed CE-wrFlu method performance was compared to previously reported chiral CE-Flu methods for AAs, which almost all employ visible laser-induced excitation [16, 38–44] (Table 3). Using FMOC, the derivatization time (2 min) was significantly shorter than for the earlier applied derivatization agents, in particular for FITC (> 12 h), i.e., the most commonly used agent. The number of enantioseparated proteinogenic AAs (16 with chiral resolution > 1.0) was also considerably higher than for previously reported methods (maximum of 8 AAs), while chiral separation of a mixture of 11 AAs could be achieved in a single run applying the currently presented method. Considering that our method employs regular lamp-based excitation, the achieved sensitivity is very satisfactory, exhibiting lower sample LODs than reported for most of the LIF-based chiral CE-Flu methods. The only chiral CE-Flu method employing UV excitation published so far [41] showed more favorable sensitivity but was developed for one AA only (aspartic acid).

**Table 3:** Comparison with previously reported chiral CE-Flu methods for AAs

Separation method and BGE	Derivatization agent and time	Excitation source and wavelength(s)	AAs with enantioresolution ≥ 1.0	Lowest LOD (injected concentration/sample concentration; nM)	Application	Ref.
MEKC; 100 mM sodium tetraborate (pH 10.0), 80 mM SDS, 20 mM β-CD	FITC; overnight	Ar+ laser; 488 nm	Arg, Ala, Glu, Asp, Ser, Leu, Gln, Lys	0.7/7	CSF	[16]
MEKC; 100 mM sodium tetraborate (pH 10.0), 80 mM SDS. 20 mM β-CD	FITC; overnight	Ar+ laser; 488 nm	Arg, Ala, Glu, Asp, Ser	160/3200	Maize	[38]
MEKC; 100 mM sodium tetraborate (pH 9.4), 30 mM SDS. 20 mM β-CD	FITC; overnight	Ar+ laser; 488 nm	Arg, Asn, Ser, Ala, Glu, Asp	0.3/1200	Orange juice	[39]
MEKC; 100 mM sodium tetraborate (pH 9.7), 30 mM SDS, 20 mM β-CD	FITC; overnight	Ar+ laser; 488 nm	Arg, Ala, Glu, Asp	16.6/8000	Vinegars	[40]
MEKC; 150 mM Tris-borate (pH 9.0), 150 mM SDS with 60 mM HP-β-CD	NDA; 20 min	Violet LED; 395–425 nm	Asp	0.25/2.5	CSF, soymilk, beer	[41]
CE; 100 mM borate (pH 8.0), 8 mM DM-β-CD and 5 mM HPA-β-CD	NBD-F; 10 min	Ar+ laser; Ex, 488 nm	Glu, Asp	50/600	Brain	[42]
MEKC; 10 mM sodium borate (pH 9.1), 12 mM SC, 1.6% HSA, 10% methanol	DTAF; 30 min	Ar+ laser; Ex, 488 nm	Glu, Asp	0.15/180	Urine	[43]
MEKC; 80 mM sodium tetraborate (pH 9.2), 30 mM γ-CD, 30 mM STC, 5% acetonitrile	CFSE; 2 h	Ar+ laser; Ex, 488 nm	Ala, Glu, Asp, His, Ser, Leu, Val	5/5	Water from Mono Lake, CA	[44]
MEKC; 40 mM sodium tetraborate (pH 9.5), 30 mM SDS, 30 mM β-CD, 15% isopropanol	FMOC; 2 min	Xe-Hg lamp; 210–300 nm	Ala, Val, Met, Thr, His, Ile, Glu, Asp, Leu, Phe, Trp, Pro, Ser, Asn, Gln, Arg	14/280	CSF	this work

*HP-β-CD* hydroxypropyl-β-cyclodextrin, *DM-β-CD* dimethyl-β-cyclodextrin, *HPA-β-CD* hydroxylpropylamino-β-cyclodextrin, *SC* sodium cholate, *HSA* human serum albumin, *DTAF* 5-(4,6-dichloro-s-triazin-2-ylamino) fluorescein, *STC* sodium taurocholate, *CFSE* 5-carboxyfluorescein succinimidyl ester, *Ala* alanine, *Arg* arginine, *Asn* asparagine, *Asp* aspartic acid, *Glu* glutamic acid, *Gln* glutamine, *His* histidine, *Ile* isoleucine, *Leu* leucine, *Lys* lysine, *Met* methionine, *Phe* phenylalanine, *Pro* proline, *Ser* serine, *Thr* threonine, *Trp* tryptophan, *Val* valine

## Conclusion

A new chiral CE method for AAs was developed encompassing fast derivatization with FMOC followed by selective separation employing a BGE with β-CD and SDS and sensitive fluorescence detection. Efficient broad-band UV excitation of FMOC-AAs was achieved using a Xe-Hg lamp in combination with a short-pass excitation filter. The optimized CE-Flu method enabled enantioseparation of 16 FMOC-dl-AAs with a resolution of 1.0 or higher. Use of wave-guiding principles for emission light collection provided good sensitivity, and further improvement of the *S*/*N* for individual enantiomers was accomplished by wavelength-resolved CCD detection utilizing signal averaging along the wavelength axis. Resulting LODs for most AA enantiomers were between 10 and 100 nM (2–15 ng/mL injected concentration), which is significantly better than the micromolar range LODs reported for similar analyses by chiral CE using UV absorbance detection [45–48]. The LODs for CE-Flu are similar to LODs obtained for FMOC-AAs using LIF detection employing a home-built UV laser [49]. Compared to chiral FMOC-AA analysis using CE-MS, LODs for CE-Flu were better with a factor of 3 for arginine and isoleucine up to a factor of 85 for valine in aqueous solution [58]. The CE-Flu LOD for d-leucine in CSF was 11.9 times lower than the LOD for this AA obtained with CE coupled with mass spectrometry (CE-MS) [58]. The new chiral CE-Flu method was demonstrated to allow separation and detection of AA enantiomers in CSF, providing sufficient resolution and sensitivity to probe low levels of endogenous d-aspartic acid and d-glutamine next to excess l-AAs.

Compared with several previously reported chiral CE-Flu methods for AAs using LIF detection, the presented method has the advantage of fast derivatization time (2 min), number of AAs enantioseparated (16 in total; 11 in one run), and favorable sensitivity while employing straightforward lamp excitation. As for most other chiral CE-Flu methods, the LODs for real samples were compromised by a dilution of the derivatized sample before injection, which was needed to avoid interferences by matrix components and overloading of the unreacted FMOC. For the presented method a 10-times dilution was adequate, whereas other methods required much higher dilutions, deteriorating the ultimate LODs [16, 38–44]. The intrinsic sensitivity of the new CE-Flu system would allow d-AA detection down to the low-nanomolar range, but that would require an improved sample cleanup.
